# Disease progression in Parkinson’s disease patients with mild cognitive impairment: 5-year longitudinal study from the early Parkinson’s disease longitudinal Singapore (PALS) cohort

**DOI:** 10.18632/aging.206040

**Published:** 2024-08-12

**Authors:** Xiao Deng, Seyed Ehsan Saffari, Bin Xiao, Samuel Yong Ern Ng, Nicole Chia, Xinyi Choi, Dede Liana Heng, Zheyu Xu, Kay-Yaw Tay, Wing-Lok Au, Eng-King Tan, Louis CS Tan

**Affiliations:** 1 National Neuroscience Institute, Singapore, Singapore; 2 Duke-NUS Medical School, Singapore, Singapore; 3 Centre for Quantitative Medicine, Duke-NUS Medical School, Singapore, Singapore

**Keywords:** Parkinson’s disease, mild cognitive impairment, disease progression

## Abstract

Aim: To investigate motor, non-motor and cognitive progression in early Parkinson’s disease (PD) patients with Mild Cognitive Impairment (MCI).

Methods: PD patients were recruited within 1 year of diagnosis and were classified into PD-MCI group and PD with normal cognition (PD-NC) group. H&Y staging scale, MDS-UPDRS part III were used to assess disease severity and motor progression. Non-motor symptom scale (NMSS) was used to evaluate the NMS progression. Cognitive progression was assessed from 5 cognitive domains. Annual progression changes in the longitudinal outcomes were examined via linear mixed model with random intercept effect. False discovery rate (FDR) method was performed to control for multiple testing comparison and q-value was calculated. We set the threshold of q-values as 0.1.

Result: A total of 205 PD patients, including 107 PD-MCI and 98 PD-NC patients were assessed prospectively over a 5-year period. PD-MCI patients, compared to PD-NC group, had a significantly higher progression rate in H&Y score (0.11 vs. 0.06, p=0.03, q=0.08), MDS-UPDRS motor score (3.11 vs. 1.90 p<0.001, q=0.06) and postural instability gait difficulty (PIGD) score (0.40 vs. 0.20, p=0.02, q=0.07). PD-MCI group also exhibited significantly faster deterioration in NMSS perceptual domain (PD-MCI vs. PD-NC: 0.38 vs. -0.04, p=0.01, q=0.06) and cognitive visuospatial domain (PD-MCI vs. PD-NC: 0.13 vs. -0.06, p=0.048, q=0.09) after adjustment for confounders and multiple comparisons.

Conclusions: PD-MCI patients had faster decline in motor functions, visuo-perceptual and visuospatial performance. These findings provide a more comprehensive prognosis of PD-MCI, which could be helpful for clinician to manage PD-MCI patients.

## INTRODUCTION

Mild cognitive impairment (MCI) denotes an intermediary stage between normal cognitive function and dementia. MCI is commonly presented in patients with Parkinson’s Disease (PD), even in the early stages. The prevalence of MCI in non-demented PD patients ranges from 19 to 55% according to different clinical setting and diagnostic criteria [1–3]. The presence of MCI in PD patients is associated with an increased risk of developing PD dementia (PDD) [4], which is linked to a poor quality of life.

Longitudinal PD-MCI studies mainly focused on depicting the cognitive trajectories or conversion but yielded heterogenous results. Previous studies have shown that 39% to 59% of the PD-MCI patients progressed to PDD in a 5-year study, while 11% to 27.8% of PD-MCI patients reverted to normal cognition over the same follow-up period [5, 6]. However, these studies have not specifically studied the progression of motor and non-motor symptoms (NMS) amongst PD-MCI patients. Recently, it was reported that PD-MCI patients experienced a swifter decline in daytime sleepiness and quality of life compared to PD patients with intact cognitive function [7]. However, this study only assessed limited NMS domains and recruited PD patients from different disease stages, possibly compromising the generalisability of the study.

To the best of my knowledge, the comprehensive profile of disease progression in PD-MCI patients remains largely unknown. In view of the scarcity of evidence in this domain, we performed a longitudinal study to evaluate the comprehensive progression of PD-MCI patients. We hypothesize that PD-MCI patients have faster motor, non-motor and cognitive progression.

## MATERIALS AND METHODS

### Study population

The prospective study involved 205 early-stage PD patients, recruited within one year of their PD diagnosis, following the Early Parkinson’s Disease Longitudinal Singapore (PALS) study protocol [8]. All the PD patients were subject to annual follow-ups for a duration of up to five years. Our study took place at two outpatient clinics specializing in movement disorders in Singapore and received approval from the SingHealth Centralized Institutional Review Board (CIRB) under Reference 2019/2433. All participants provided written informed consent prior to participation. PD patients were categorized into two groups: PD-MCI and PD patients with normal cognition (PD-NC), according to the Movement Disorder Society (MDS) Task Force Level II diagnostic criteria [4]. Based on this criterion, cognitive impairment ought to be observable in a minimum of two neuropsychological tests, where scores deviate by 1.5 standard deviations (SDs) below established standard [9], either within one single cognitive domain or across various domains.

### Data collection

Demographic data were collected from all patients and comprehensive profiles of the disease progression including motor, non-motor and cognitive domains were examined. All clinical assessments were conducted while patients were on their PD medications. The assessments were evaluated by the specialist at baseline and follow-up visits. The follow-up visits were performed on an annual basis, up to 5 years. Standardized formula was used to calculate the levodopa-equivalent daily dose (LEDD) [10]. Modified Hoehn and Yahr (H&Y) staging scale, Movement Disorder Society-Unified Parkinson’s Disease Rating Scale (MDS-UPDRS) part III motor score were used to assess disease severity and motor conditions, respectively. MDS-UPDRS motor sub scores (Postural Instability and Gait Disorder (PIGD) score and tremor score) were calculated [11]. We evaluated the overall NMS burden by Non-motor symptom scale (NMSS) [12], which consists of 9 different territories (cardiovascular, sleep/fatigue, mood/apathy, perceptual problems/hallucinations, attention/memory, gastrointestinal, urinary, sexual function and miscellaneous). Rapid Eye Movement (REM) Sleep Behaviour Disorder (RBD) was evaluated by the RBD Single-Question Screen (RBD1Q) [13]. Overall cognitive change was evaluated by Montreal Cognitive Assessment (MoCA) score [14]. Five different cognitive domains were assessed via 10 neuropsychological tests. Specifically, Frontal Assessment Battery (FAB) [15] total score and Fruit Fluency tests were used in Executive domain; Judgment of Line Orientation from the Repeatable Battery for the Assessment of Neuropsychological Status (RBANS) [16] and the total score from the copy task of the Rey-Osterrieth Complex Figure Test (ROCF) [17] were performed to evaluate visuospatial domain; the delayed recall scores from both the Alzheimer Disease Assessment Scale (ADAS-cog) and the ROCF for memory domain; the total scores of the Digit Span Backward and Symbol Span tests for Attention and working memory domain; finally, the total scores from the Boston Naming Test (BNT) and the Similarities subtest of the Wechsler Adult Intelligence Scale | Fourth Edition (WAIS-IV) were utilized in the Language domain. The scores for the five cognitive domains (executive function, visuospatial abilities, memory, attention, and language) were computed by averaging the standardized scores (Z-scores) from two neuropsychological tests within each respective domain.

### Statistical analysis

Data processing and statistical analysis were performed using Stata/SE 16 and SAS OnDemand for Academics (SAS Institute Inc. 2014). Continuous variables were summarized using either the mean and standard deviation (SD), or the median along with the first and third quartiles, where appropriate. Frequency counts and percentages were employed to depict categorical variables. Two-sample t test or Mann–Whitney U test (depending on the tenability of the normality assumption) was conducted to compare continuous variables, while Fisher’s exact test was utilized to compare the categorical variables between the two groups.

The longitudinal outcomes trend spanning 5 years, from baseline to follow-up visits, was investigated using a longitudinal, linear mixed model. This model was adjusted for potential confounders such as age, sex, and years of education. The disease progression slopes between the two groups (PD-MCI vs. PD-NC) were estimated by random intercept-only model with an unstructured covariance matrix and a residual pseudo-likelihood method. The slope estimate, including the beta coefficient and its corresponding 95% confidence intervals, were provided. The normality assumption was evaluated using standardized residuals. All outcomes in the study are exploratory with no prior hierarchical hypothesis. Significance level was set at p < 0.05. The false discovery rate (FDR) method [18] was employed to manage multiple testing comparisons, and q-values were computed accordingly. We established a threshold for q-values at 0.1.

### Data availability statements

The datasets produced and/or analysed during the present study are not publicly accessible due to ethical constraints. However, they can be obtained from the corresponding author upon reasonable request.

## RESULTS

### Demographic and clinical features of patients

During a 5-year period, a prospective assessment was conducted on a total of 205 patients with PD, comprising 107 PD-MCI and 98 PD-NC patients. At the baseline, there were no notable discrepancies observed in terms of ethnicity, sex, H&Y scale, RBD1Q, and NMSS score between the two groups. Compared to the PD-NC group, PD-MCI group had an older age of diagnosis and fewer years of education. The summary of the patients’ demographic and clinical characteristics at baseline is found in Table 1. The number of patients in the follow-up visits is shown in Supplementary Table 1.

**Table 1:** Demographic and clinical characteristics at baseline.

	**PD-NC**	**PD-MCI**	**†p-value**
	**n=98**	**n=107**	
Ethnicity: Chinese	87 (88.78%)	89 (83.18%)	0.51
Age of onset (year)	60.0 ± 9.6	65.3 ± 8.0	<0.001*
Age of diagnosis (year)	60.9 ± 9.4	65.8 ± 8.0	<0.001*
Sex: male	60 (61.2%)	61 (57.0%)	0.57
Education (Year)	12.0 (10.0, 16.0)	10.0 (6.0, 10.0)	<0.001*
H&Y scale	2.0 (1.5, 2.0)	2.0 (1.5, 2.0)	0.058
NMSS Total Score	14 (8, 24)	14 (9,29)	0.45
RBD1Q	0.63(0.3,1.0)	0.64(0.3, 1.0)	0.98
LEDD at baseline	184.8 ± 142.8	202.1 ± 133.2	0.39
LEDD at year 2	235.6 ± 133.5	250.4 ± 121.6	0.56
LEDD at year 3	304.8 ± 113.2	310.6 ± 123.4	0.23
LEDD at year 4	365.6 ± 123.5	380.4 ± 130.2	0.27
LEDD at year 5	443.6 ± 136.5	456.3 ± 144.6	0.18

Abbreviations: H&Y, Modified Hoehn and Yahr (H&Y) staging scale; NMSS, non-motor symptom scale; RBD1Q, Rapid Eye Movement Sleep Behaviour Disorder Single-Question Screen; LEDD, Levodopa equivalent daily dose.

Categorical variables reported as frequency (%); continuous variables reported as mean ± standard deviation or median and first and third quartile (where appropriate).

†Fisher exact test and two-sample t test or Mann Whitney U test (where appropriate) for categorical and continuous variables, respectively.

### Longitudinal progression of motor, NMS and cognitive functions

**Figure 1:**
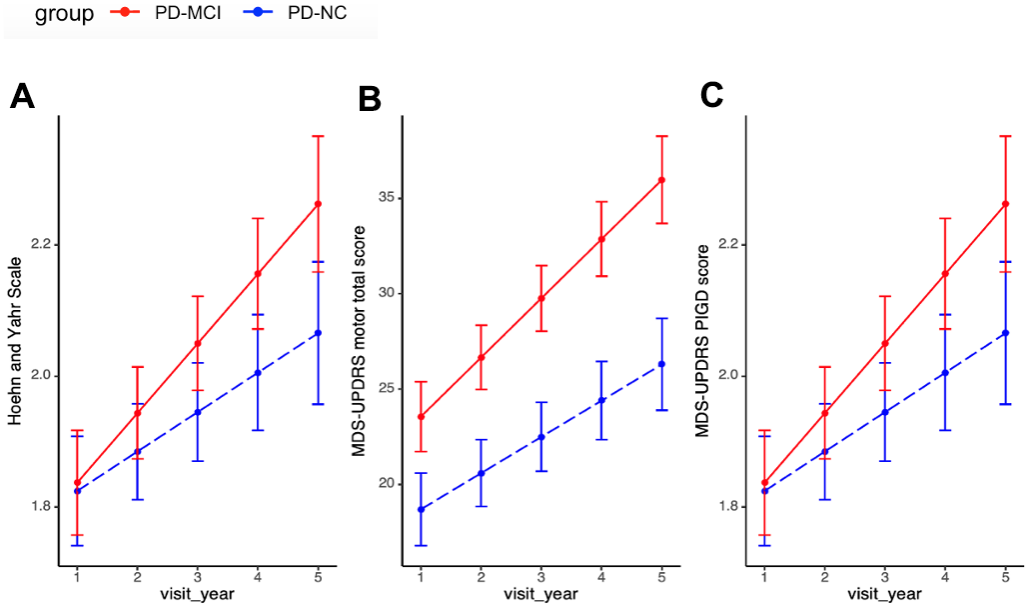
**Motor progression between PD-MCI and PD-NC.** (**A**) Progression slopes of H&Y score in PD-MCI and PD-NC group were 0.11 vs. 0.06, p=0.03. (**B**) Progression slopes of UPDRS motor score in PD-MCI and PD-NC group were 3.11 vs. 1.90 p=0.00. (**C**) Progression slopes of PIGD score in PD-MCI and PD-NC group were 0.45 vs. 0.24, p=0.02. Longitudinal linear mixed model was performed to compare the progression slopes of motor outcomes between PD-MCI and PD-NC group; analysis has been adjusted for age of diagnosis, sex and education year. Abbreviations: H&Y: Modified Hoehn and Yahr staging scale; MDS-UPDRS: Movement Disorder Society-Unified Parkinson’s Disease Rating Scale; PIGD: Postural Instability and Gait Disorder.

Overall, PD-MCI groups had worse motor progression in the 5-year longitudinal study. Specifically, PD-MCI patients experienced a notably accelerated rate of progression in H&Y score, MDS-UPDRS motor score and PIGD score than PD-NC adjusted for age of diagnosis, sex and education years. The faster motor progression rate in PD-MCI patients remained statistically significant following adjustment for multiple comparisons (0.11 vs. 0.06, p=0.03, q=0.08; 3.11 vs. 1.90 p=0.00, q=0.06; 0.45 vs. 0.24, p=0.02, q=0.07, respectively, Table 2 and Figure 1).

**Table 2:** Comparison of motor, NMS and cognitive progression rates between two groups.

**Longitudinal outcomes**	**Group**	**Slope estimate**	**95% CI**		**†p-value**	**††q-value**
			**Lower**	**Upper**		
**Motor**						
H & Y scale	PD-NC	0.06	0.03	0.09	0.03*	0.08*
	PD-MCI	0.11	0.08	0.14		
MDS-UPDRS motor score	PD-NC	1.9	1.3	2.51	0.005*	0.06*
	PD-MCI	3.11	2.53	3.68		
PIGD Score	PD-NC	0.2	0.09	0.32	0.02*	0.07*
	PD-MCI	0.4	0.29	0.51		
**Non-motor**						
NMSS total score	PD-NC	2.06	0.61	3.49	0.14	0.26
	PD-MCI	3.74	1.99	5.49		
NMSS domain 4 score	PD-NC	-0.04	-0.24	0.16	0.01*	0.06*
	PD-MCI	0.38	0.14	0.61		
**Cognitive**						
Memory score	PD-NC	0.1	0.06	0.13	0.73	0.73
	PD-MCI	0.09	0.04	0.13		
Visuospatial score	PD-NC	-0.06	-0.1	-0.01	0.048*	0.09*
	PD-MCI	-0.13	-0.18	-0.08		
Attention score	PD-NC	0.04	-0.01	0.08	0.22	0.30
	PD-MCI	-0.01	-0.06	0.04		
Executive score	PD-NC	-0.03	-0.06	0.00	0.34	0.42
	PD-MCI	-0.05	-0.09	-0.02		
Language score	PD-NC	0.03	-0.01	0.06	0.38	0.42
	PD-MCI	0.05	0.01	0.08		
MoCA score	PD-NC	-0.21	-0.37	-0.05	0.21	0.30
	PD-MCI	-0.36	-0.54	-0.18		

Abbreviations: H&Y, Modified Hoehn and Yahr staging scale; MDS-UPDRS, Movement Disorder Society-Unified Parkinson’s Disease Rating Scale; PIGD, Postural Instability and Gait Disorder; NMSS, non-motor symptom scale; NMSS domain 4 (perceptual problems/hallucinations); MoCA score: Montreal Cognitive Assessment score.

†The trend of longitudinal motor outcomes from baseline to follow-up visits were examined by means of longitudinal, linear mixed model, adjusting for age, sex and education year.

††False discovery rate (FDR) method was performed and q-values were calculated to control for multiple testing and the threshold of q-values was set as 0.1.

**Figure 2:**
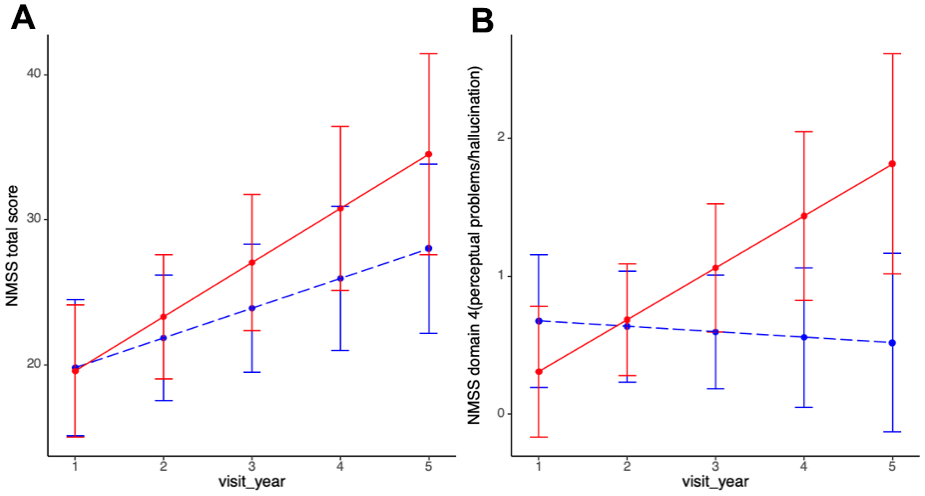
**Non-motor progression between PD-MCI and PD-NC.** (**A**) Progression slopes of NMSS total score in PD-MCI and PD-NC group were 3.74 vs. 2.06, p=0.14. (**B**) Progression slopes of NMSS domain 4 score in PD-MCI and PD-NC group were 0.38 vs. -0.04, p=0.01. Longitudinal linear mixed model was performed to compare the progression slopes of non-motor outcomes between PD-MCI and PD-NC group; analysis has been adjusted for age of diagnosis, sex and education year. Abbreviations: NMSS: non-motor symptom scale; NMSS domain 4 (perceptual problems/hallucinations).

In terms of NMS progression, both groups showed no significant differences in changes in NMSS total score. However, in the further analysis of NMSS domain scores, PD-MCI group had significant more rapid progression rate in NMSS domain 4 (perceptual problems/hallucination) after adjusting for age of diagnosis, sex, education years and multiple comparisons (PD-MCI vs. PD-NC: 0.38 vs. -0.04, p=0.01, q=0.06, Table 2 and Figure 2).

**Figure 3:**
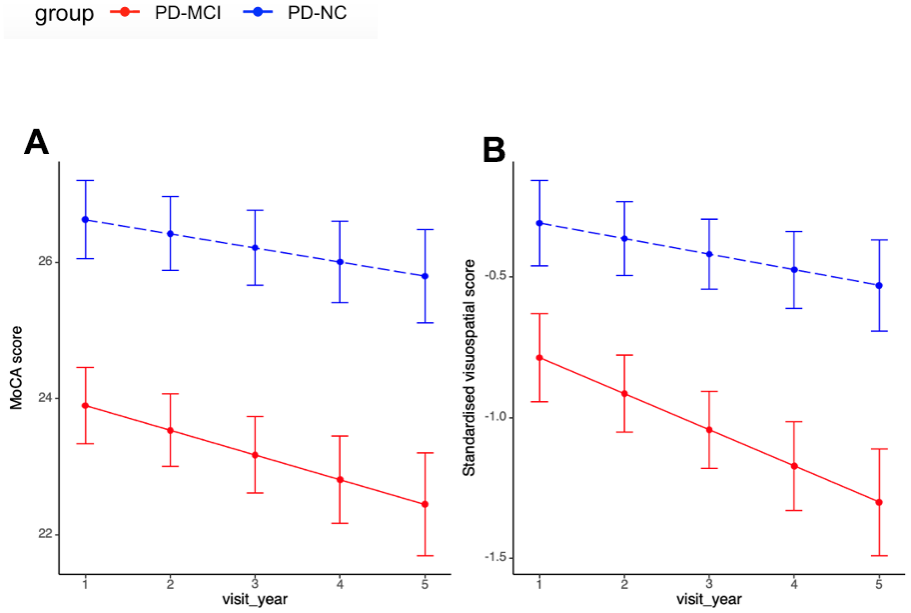
**Cognitive progression between PD-MCI and PD-NC.** (**A**) Progression slopes of MoCA score in PD-MCI and PD-NC group were -0.36 vs. -0.21, p=0.21. (**B**) Progression slopes of standardised visuospatial score in PD-MCI and PD-NC group were -0.13 vs. -0.06, p=0.048. Longitudinal linear mixed model was performed to compare the progression slopes of cognitive outcomes between PD-MCI and PD-NC group; analysis has been adjusted for age of diagnosis, sex and education year. Abbreviations: MoCA score: Montreal Cognitive Assessment score.

With regards to overall cognitive progression, statistically notable difference was not observed in MoCA score between PD-MCI and PD-NC group. However, in the additional analysis of cognitive domain progression, PD-MCI group exhibited significantly faster deterioration in visuospatial domain after adjustment for cofounders and multiple comparison (PD-MCI vs. PD-NC: -0.13 vs. -0.06, p=0.048, q=0.09, Table 2 and Figure 3). PD-MCI group also showed a more rapid but insignificant decline in attention and executive domains.

## DISCUSSION

In this 5-year prospective study, we comprehensively investigated the motor, non-motor and cognitive progression of PD-MCI patients as compared to PD-NC patients. PD-MCI patients had greater deterioration in motor, visuo-perceptual and visuospatial performances. Specifically, patients with PD-MCI exhibited markedly greater progression rates in H&Y scores, MDS-UPDRS motor scores, PIGD scores, NMSS perceptual problems/hallucinations domain scores, and standardized visuospatial scores (cognitive domain).

The current literature indicates that motor symptoms have an impact on cognition [19, 20]. PD patients with akinetic-rigid subtype are associated with faster cognitive decline and greater risk of dementia [20]. However, it is largely unknown whether PD-MCI patients have faster motor progression. Currently there is only one longitudinal study with an average follow-up time of 2.4 years that assessed motor progression in PD-MCI patients. The study showed that PD-MCI patients had similar MDS-UPDRS motor progression rates compared to PD-NC group [7]. On the contrary, we found that PD-MCI patients had significantly faster progression rate in all motor functions except the tremor scores. Several factors might account for the discrepancy. Firstly, all our PD patients were diagnosed within 1 year from their diagnosis, while the recent study recruited PD patients from different disease stages. Secondly, all our motor assessments were performed during the “on” period, while the recent study examined the motor functions during off period. Lastly, our study had a longer follow-up period. Despite the challenge of inferring causality, our study demonstrated that PD-MCI patients indeed exhibit faster motor progression. For PD-MCI patients, clinicians and researchers tend to focus more on their cognitive conversion or trajectories but not motor progression. However, in view of our findings, closer management of motor progression is also warranted in PD-MCI patients.

To the extent of my understanding, this study represents the initial longitudinal investigation that comprehensively assessed the NMS progression using NMSS in PD-MCI patients. We found that PD-MCI patients exhibited a significantly more rapid decline in NMSS perceptual problems/hallucinations domain than the PD-NC group. We also found that PD-MCI patients had significantly higher progression rate in standardized visuospatial score (one of the cognitive domains), which was in line with the findings from a recent study [7]. The PD-MCI group demonstrated a notably older age at diagnosis in comparison to the PD-NC group. While statistical analysis has been performed with adjustment for age, the age difference may still be a contributing factor to the observed poorer prognosis in the PD-MCI group. Different pathways are involved in PD-MCI and the detailed mechanism of PD-MCI is not yet clear. The deterioration in NMSS domain 4 (perceptual problems/hallucinations) and standardized visuospatial score in PD-MCI patients suggests that they might share a similar neurobiological basis. It has been reported that PD-MCI patients with visuo-perceptual and visuospatial changes were associated with cortical thinning in posterior regions in structural magnetic resonance imaging (MRI) in a 4-year follow-up study [21]. The faster visuo-perceptual and visuospatial deterioration in PD-MCI patients may be also related to the posterior cortical impairment due to Lewy body deposition influenced by MAPT genotype [22]. It may be helpful to explore the underlying mechanism by genotyping relevant SNPs in PD-MCI patients. A previous study found that PD-MCI patients demonstrated significant progression in attention and executive functions [7]. In our study, we observed a faster progression trend in these two cognitive domains among PD-MCI patients, although it did not achieve statistical significance, which could be attributed to the limited sample size in our study.

As a longitudinal study with comprehensive analysis of progression in PD-MCI patients, our study has several strengths. Initially, the study was conducted prospectively in a homogeneous Asian cohort, with all PD patients recruited within one year of diagnosis. Variances in disease stages, one of the main confounders for progression, can therefore be avoided. Second, NMSS and detailed neuropsychological assessments allow us to obtain a comprehensive picture of NMS. Nevertheless, it’s important to acknowledge certain limitations of the study. This is a single-cohort study with a restricted sample size, necessitating further validation in diverse populations. This study is an ongoing prospective longitudinal study. Seventy patients had reached the fifth-year visit time-point. Approximately 5% of individuals in the PD-NC group progress to PD-MCI, while a comparable proportion of PD-MCI patients revert to the PD-NC group. This dynamic maintains the overall stability of participant numbers in each group during follow-up visits. RBD is an independent risk factor for cognitive impairment in PD [23]. However, RBD was not significant different between two groups in our cohort, which was likely due to the utilization of RBD1Q for assessing RBD instead of the gold standard overnight polysomnography assessment. In addition, the interaction effect between motor and visuospatial performance cannot be ruled out completely as motor dysfunctions could potentially compromise the validity of some neuropsychological tests [21]. However, we mitigated this by selecting assessments that were less dependent on time-based movements and ensured that these were performed in the medication “on” state.

In conclusion, our results indicate that PD-MCI patients experienced a more rapid decline in motor function, visuo-perceptual abilities, and visuospatial performances. These findings offer a comprehensive perspective of how patients with PD-MCI progress that will be beneficial in aiding clinicians in managing PD-MCI patients. Further validation of the progression of PD-MCI patients in larger cohorts is warranted.

## Supplementary Material

Supplementary Table 1
