# Supplementary material for: Disease progression in Parkinson’s disease patients with mild cognitive impairment: 5-year longitudinal study from the early Parkinson’s disease longitudinal Singapore (PALS) cohort
Source: Aging (Albany NY). 2024 Aug 12;16(15):11491–500. doi: 10.18632/aging.206040 (PMC11346777; doi:10.18632/aging.206040)
Supplement: Supplementary Table 1 [file aging-16-206040-s001.pdf]

SUPPLEMENTARY TABLE

Supplementary Table 1. Number of patients in the follow-up (%).

Visit	overall (n)	PD-MCI(n,%)	PD-NC(n,%)
Baseline	205	107(100%)	98(100%)
2nd Year	172	91(85.0%)	81(82.7%)
3rd Year	138	73(68.2%)	65(66.3%)
4th Year	116	65(60.7%)	51(52.0%)
5th Year	70	35 (32.7%)	35(35.7%)
